# Electrical Resistivity of Cu and Au at High Pressure above 5 GPa: Implications for the Constant Electrical Resistivity Theory along the Melting Curve of the Simple Metals

**DOI:** 10.3390/ma14195476

**Published:** 2021-09-22

**Authors:** Innocent C. Ezenwa, Takashi Yoshino

**Affiliations:** 1 Institute for Planetary Materials, Okayama University, 827 Yamada Street, Misasa, Tottori 682-0193, Japan; tyoshino@misasa.okayama-u.ac.jp; 2 Now at the Earth and Planets Laboratory, Carnegie Institute for Science, Washington, DC 20015, USA

**Keywords:** electrical resistivity, thermal conductivity, electrons and phonons interactions, high pressure and temperature, constant resistivity, melting curve

## Abstract

The electrical resistivity of solid and liquid Cu and Au were measured at high pressures from 6 up to 12 GPa and temperatures ∼150 K above melting. The resistivity of the metals was also measured as a function of pressure at room temperature. Their resistivity decreased and increased with increasing pressure and temperature, respectively. With increasing pressure at room temperature, we observed a sharp reduction in the magnitude of resistivity at ∼4 GPa in both metals. In comparison with 1 atm data and relatively lower pressure data from previous studies, our measured temperature-dependent resistivity in the solid and liquid states show a similar trend. The observed melting temperatures at various fixed pressure are in reasonable agreement with previous experimental and theoretical studies. Along the melting curve, the present study found the resistivity to be constant within the range of our investigated pressure (6–12 GPa) in agreement with the theoretical prediction. Our results indicate that the invariant resistivity theory could apply to the simple metals but at higher pressure above 5 GPa. These results were discussed in terms of the saturation of the dominant nuclear screening effect caused by the increasing difference in energy level between the Fermi level and the *d*-band with increasing pressure.

## 1. Introduction

The investigation of the electrical transport in transition metals at extreme pressure and temperature conditions is generally of interest in condensed matter physics and has a significant application in the study of deep interior planetary research since the terrestrial planetary core composition is predominately Fe [1], a member of the transition metals. At 1 atm, the electronic structure of the transition metals has been widely studied both experimentally and theoretically (e.g., [2,3,4]). Electrical resistivity investigation can probe the change in electronic structure and the dynamics of solid and liquid metals at extreme conditions (See [5] and ref. therein). The combined effects of pressure and temperature on the electrical resistivity of a transition metal are usually antagonistic resistivity decreases and increases with increasing pressure and temperature, respectively. On melting, electrons in a metal can be characterized as free particles with a short-range-order structure to first approximation [6,7,8]. Along the respective melting boundaries of the simple transition metals, the thermodynamic model [9,10] hypothesized that the opposing effects of pressure and temperature on the resistivity will offset each other in such a way that the resistivity value remains constant. We note that the word “simple transition metal” is used to describe those transition metals with a filled *d*-band state (Cu, Ag, and Au). Recently, the experimental resistivity investigation of the solid and liquid states of the simple metals at fixed pressure up to 5 GPa (Cu: [11]; Ag: [12]; Au: [13]) demonstrated that their pressure-dependent resistivity decreases along their melting curves in contrast with the theoretical prediction [9,10]. Although the above-mentioned experimental results contradict theoretical prediction, if this theory is confirmed to be true at much higher pressure, this could be a practical approach for laboratory studies. As such, the resistivity at the melting curve at an achievable pressure could serve as a proxy for the resistivity value along the melting curve at pressures that are difficult to reach experimentally. Moreover, since pressure promotes *s* to *d* electron state occupancy [14,15,16,17]), this could suggest that at very high pressure the transition metals with partially filled d-band could have their *d*-band filled and become simple metal-like. If confirmed for both filled and unfilled *d*-band transition metals, this theory could be a universal theory that applies generally to transition metals, especially to the late transition metals. The pressure value at which a given metal achieves the constant resistivity state along its melting curve could characterize the transition metal or a group of transition metals.

In this study, we investigated experimentally the electrical resistivity of solid and liquid Cu and Au at a fixed pressure between 6 and 12 GPa up to ~150 K into the liquid. We also measured their resistivity at room temperature up to ~16 GPa. Finally, the obtained results will be discussed in terms of the saturation of the dominant nuclear screening effect caused by the increasing energy level separation between the Fermi level and the *d*-band as a function of pressure.

## 2. Experimental Details

**Figure 1:**
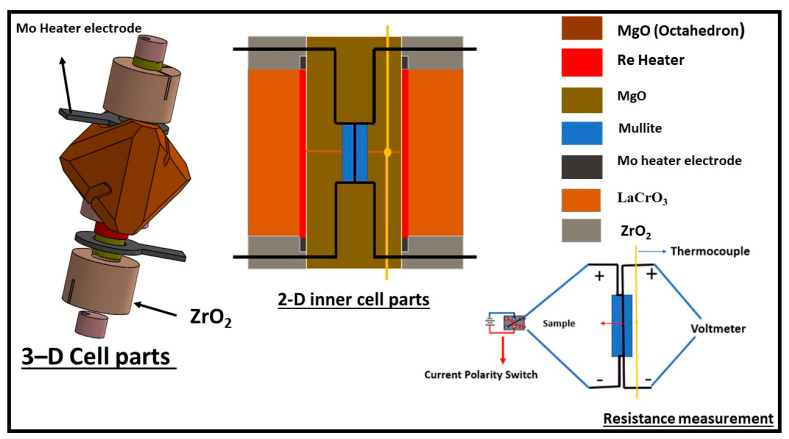
The 3D SolidWork sketch of the pressure cell assembly and the 2D sketch of the inner parts of the assembly alongside the resistance measurement setup. Modified from Ezenwa and Yoshino 2020a [19].

**Figure 2:**
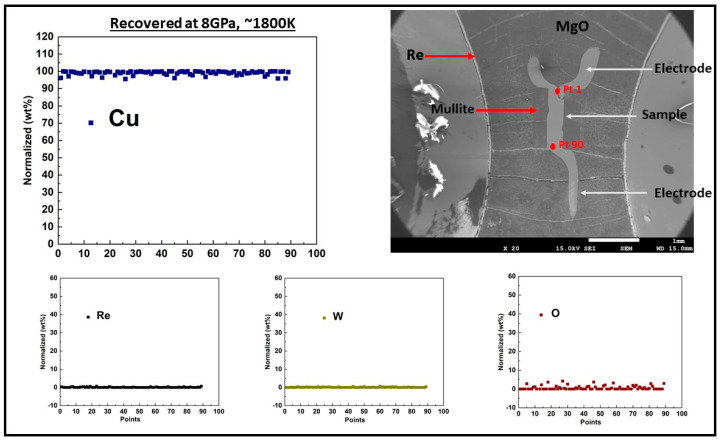
Backscattered electron (BSE) image of ex−situ recovered Cu sample at 8 GPa and ∼1800 K along with a plot of line profile of electron microprobe (EPMA) results. The line from point 1 to 90 was divided into units and each unit measured about 10–14 µm. The fourth electrode was lost during sectioning to expose the center part of the sample.

**Figure 3:**
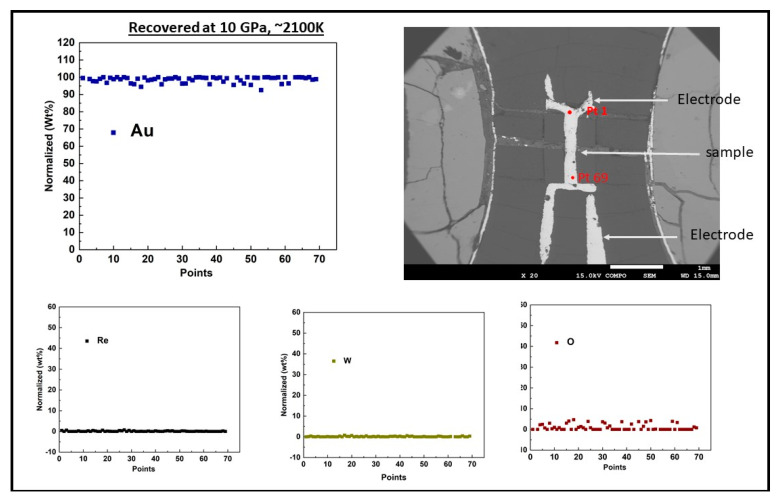
Backscattered electron (BSE) image of ex−situ recovered Au sample at 10 GPa and ∼1800 K along with a plot of line profile of electron microprobe (EPMA) results. The line from point 1 to 69 was divided into units and each unit measured about ~10–14 µm.

Measurements carried out at room temperature with increasing pressure were performed with a 10/4 pressure cell. The 10/4 cell is the same with that adopted in our earlier publication on Fe [18], with the exclusion of the heater circuit. For the measurements at high temperature at fixed pressure, we adopted an octahedron 14/6 pressure-cell with the inclusion of a heater circuit. We implemented a 4-wire resistance measurement technique [19] in both cells. A sketch of the cell design and the cell parts are shown in Figure 1. The starting dimensions of the sample wire (99.9% purity, Nilaco Corp, Tokyo, Japan) were 1.2–1.3 mm in length and 0.25 mm in diameter. The temperature of the sample encapsulated in mullite (alumina with silica composition) tube and surrounded by a baked MgO sleeve was increased by resistive heating through the passage of a high alternating current through a cylindrical Re heater. The LaCrO_3_ sleeve that surrounds the heater and the ZrO_2_ sleeve placed at the top and bottom of the heater provided thermal insulation. For the resistance measurement of the sample, a current polarity reversal switching was used to eliminate associated biased voltage mainly originating from thermal current caused by temperature gradient [20]. The electrodes and the sample were made of the same composition and the thermocouple was placed close but not in contact with the sample. To avoid the mechanical breakage of the thermocouple during compression and/or heating, the lead wires taken through the gasket were protected with a coil made of the same thermocouple composition [19]. Direct current power supply (hp E3631A) provided a constant current of 0.2 A and the sample voltage drop at fixed pressure and increasing temperature conditions were measured using a Keysight 34972A data acquisition meter (Santa Rosa, CA, USA). The captured real time graphs and the acquired raw data at fixed pressure of 10 and 6 GPa for Cu and Au, respectively, are shown in the Supplementary Section (Figures S1 and S2). The acquired data were processed to compute sample resistance using Ohm’s law, R = V/I, where R is the resistance, V is the voltage drop and I is the constant current. The ex-situ recovered sample backscatter electron (BSE) image and chemical composition analyses shown in Figure 2; Figure 3 were obtained with a scanning electron microscope (SEM) JSM-7001F and electron probe micro analyzer (EPMA) JXA-8800 at the Institute for Planetary Materials, Okayama University (Tottori, Japan) using an accelerating voltage of 15 kV and a beam current size of 12 × 10^−9^ A and 3 µm in diameter. The compositional analyses of the samples showed no trace of contamination by either sample capsule (mullite), nor the thermocouple positioned close to the sample. The ex-situ sample dimensions were determined using electron microscopy. The calculated average values of the sample length and diameter were used as an input in the calculation of the sample resistivity using Pouillet’s law ρ = RA/L, where L and A are sample length and cross-sectional area, respectively. We note that due to the sample small area relative to its large length, our current lines of flux were largely constrained in the length direction. This is important because an angular dispersed current with components in radial and length directions would require precautions in computing the sample resistivity. Using the standard error propagation technique, the error on the sample resistivity was calculated from the uncertainty in the measured geometry of the recovered sample, along with the standard deviation of the measured voltage drop across the sample at 25 K intervals in the solid and at about 20 K interval in the liquid state.

## 3. Results

### 3.1. Temperature-Dependent Electrical Resistivity of Cu and Au at a Fixed Pressure

**Figure 4:**
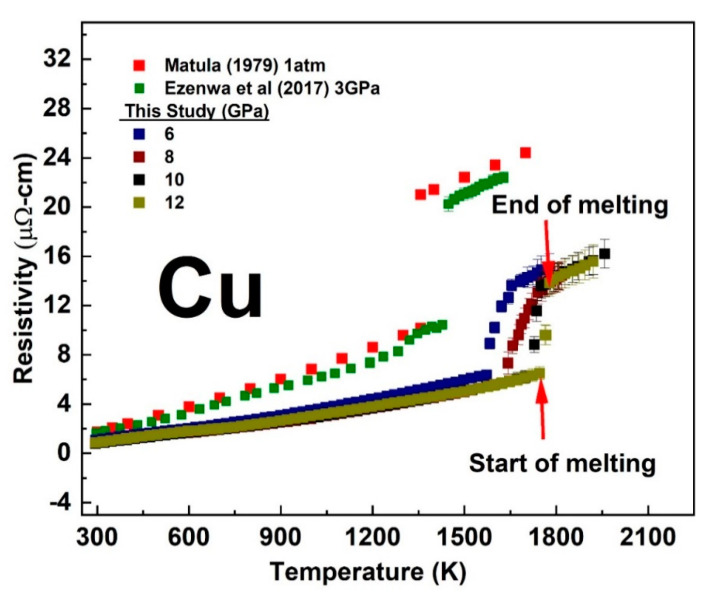
The temperature dependent electrical resistivity of Cu measured at fixed pressure in comparison with previous studies [10,19].

**Figure 5:**
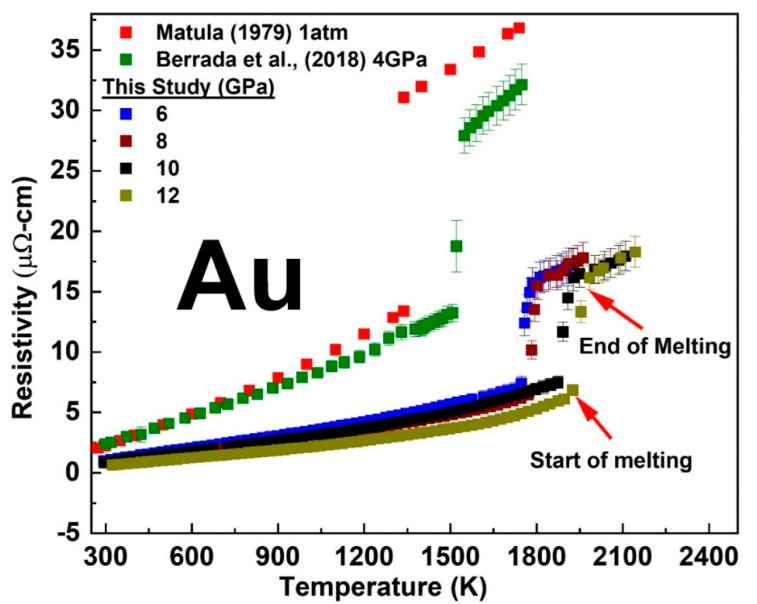
The temperature dependent electrical resistivity of Au at fixed pressure in comparison with previous studies [12,19].

The temperature-dependent electrical resistivity of solid and liquid Cu and Au measured at each fixed pressure was from 6 to 12 GPa and compared with previous studies performed at 1 atm [21] and at relatively lower pressure [11,13] are shown in Figure 4 and Figure 5. See the Supplementary Section (Figures S3 and S4) for a single plot of the temperature dependent resistivity at each fixed pressure. With increasing temperature, the resistivity of Cu and Au increased both in the solid and liquid states with a slope comparable to their 1 atm data. The linear solid-state resistivity trend with increasing temperature at each fixed pressure was fitted with the electron–phonon scattering model of the Bloch–Grüneisen function (See Supplementary Figures S5 and S6). The Bloch–Grüneisen formula is expressed as (e.g., See [22]):

$$\rho_{T}=A{\left[\frac{T}{\theta_{R}}\right]}^{n}\int_{0}^{\frac{\theta_{R}}{T}}\;\frac{x^{n}}{\left(e^{x}-1\right)\left(1-e^{x}\right)}dx$$

where ρ_(T)_ is the resistivity dependence on temperature, θ_R_ is the Debye characteristics temperature, and T is the sample temperature. Debye temperature varies with pressure in both solid and liquid materials (e.g., [23]). We are not aware of any study that has evaluated this quantity as a function of pressure for Cu and Au. Hence, we adopted its 1 atm value at higher pressure and we note that our approach was ad hoc. The value of “n” can be an integer value ranging from 1 to 5 that depends on the nature of interaction. The value of “n” can be reliably determined in relation to the low-temperature small-angle electron–phonon scattering processes that dominates at temperature below the Debye temperature. The minimum measured temperature in this study was 300 K, against 1atm Debye temperatures of ∼343 K and ∼170 K for Cu and Au respectively. At 1 atm and high temperature above Debye temperature the simple metals usually had an “n” value of ∼5 [24].

“A” is proportional to $\frac{\lambda W_{D}}{{{(}_{W_{P}})}^{2}}$; where λ is the coupling constant, *W_D_* is the Debye frequency, and *W_P_* is the Plasma frequency, See [25] for more information. By setting “n” equal to 5 in the evaluation of the Bloch–Grüneisen function, we were unsuccessful in getting a solution to our fit. This could suggest that the Debye temperature could have a strong dependence on pressure. Allowing ‘A’ and ‘n’ to vary yielded values at each fixed pressure as tabulated in Table 1 and Table 2.

**Figure 6:**
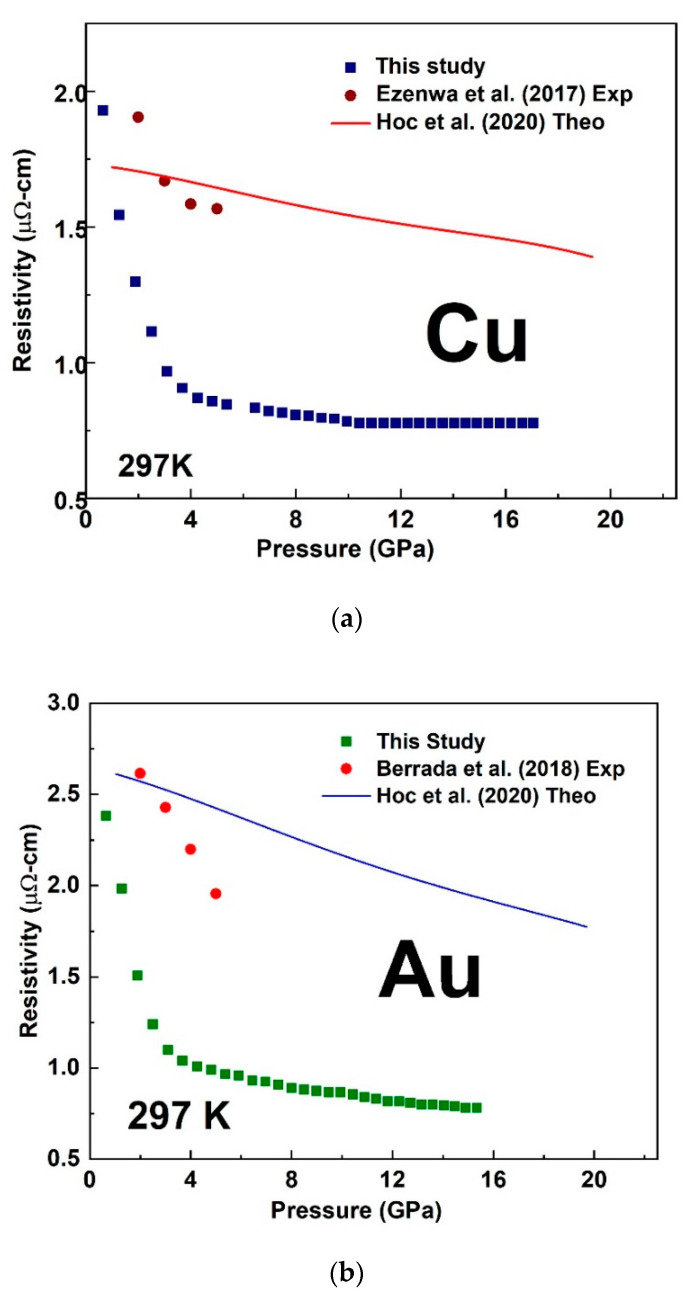
(**a**) is the pressure dependent resistivity of Cu measured up to 17 GPa, while (**b**) is that of Au measured up to 16 GPa at room Temperature compared with experimental [10,12] and theoretical [20] data.

**Figure 7:**
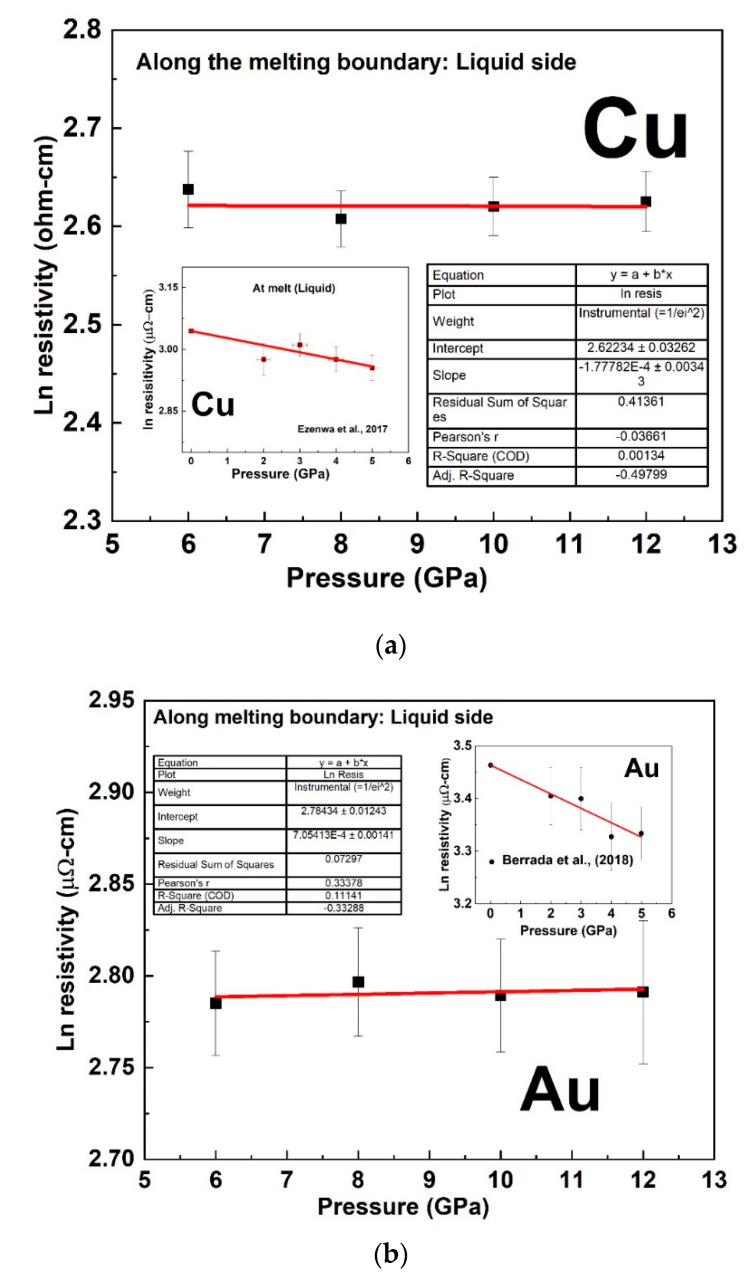
The natural logarithm of the electrical resistivity of Cu (**a**) and Au (**b**) along the pressure-dependent melting boundary. The fit line has a slope of (−1.77782 × 10^−4^ ± 0.00344) GPa−1 for Cu and (7.05413 × 10^−4^ ± 0.00141) GPa−1 for Au. The inset lower pressure data are from [10,12] for Cu and Au respectively.

The sharp change in the trend of our measured resistivity marked the start of the melting transition while its end was marked by a subsequent change in the slope above the sharp rise. At room temperature, we measured the pressure-dependent resistivity of Cu and Au up to ~16 GPa as shown in Figure 6. We observed a sharp reduction in the magnitude of resistivity versus pressure slope at ∼4 GPa in both Cu and Au (Figure 6). In comparison with experimental studies measured up to 5 GPa [11,13] and theoretical study [26], our results were slightly lower in values by about 1 µohm-cm The deviation with these experimental studies could be attributed to error in the determination of sample geometry from the ex-situ recovered samples as well as pressure calibration. However, the slope of the pressure dependence measured up to ∼4 GPa in our present study was comparable with previous studies. A best fit to the experimental data of ln resistivity versus pressure along the melting curve (See Figure 7) gave a slope (−1.77782 × 10^−4^ ± 0.00344) GPa−1 for Cu and (7.05413 × 10^−4^ ± 0.00141) GPa−1 for Au in agreement with the theoretical prediction of constant resistivity [9,10] within experimental uncertainty.

### 3.2. The Melting Curve of Cu and Au

**Figure 8:**
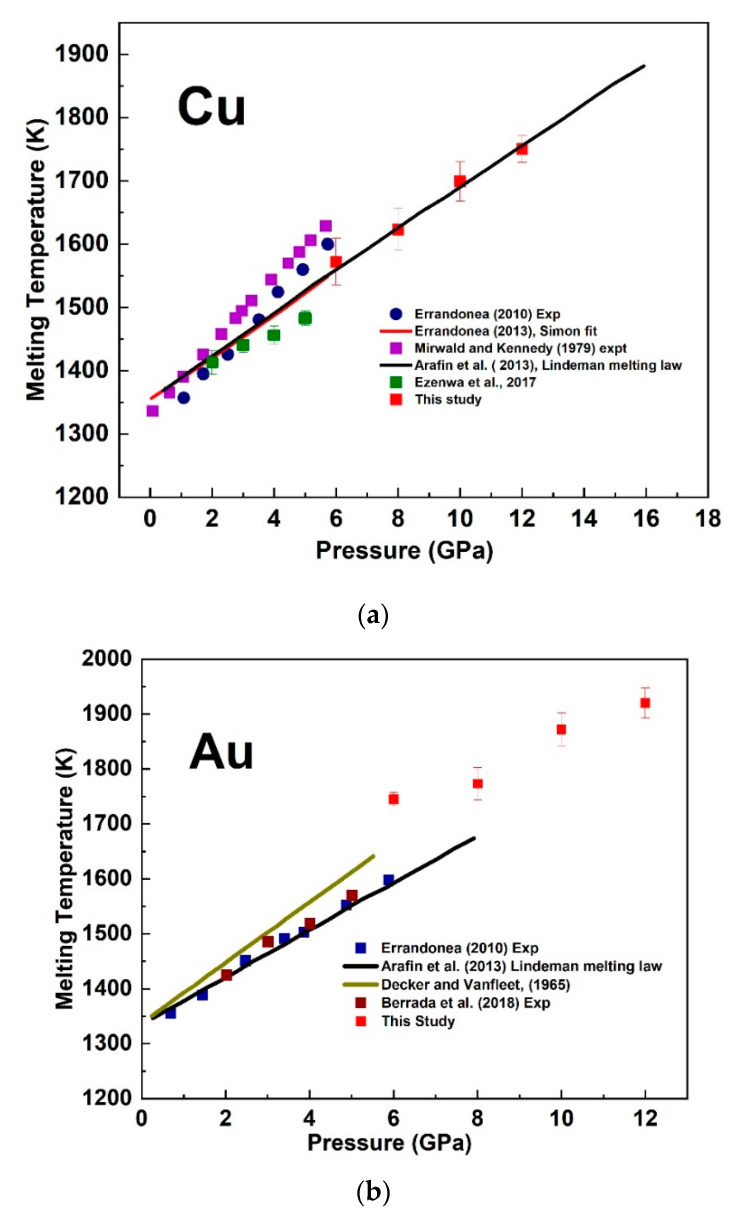
Melting temperature of Cu (**a**) and Au (**b**) as a function of pressure compared with previous studies. The melting temperature at fixed pressure was taken as the recorded temperature at the onset of melting.

A change in the electrical resistivity can probe the melting transition of metals and materials because of the abrupt response to solid–liquid phase transformation [27]. Because of the adoption of a metal heater in our cell design, there could be a temperature gradient along the sample due to the high thermal conductivity of metals. The thermocouple was positioned at the center of the sample, the hottest part of the sample. At each fixed pressure in our measurement, we determined the melting temperature as the corresponding temperature at the onset of melting. The estimated error came from the average value of the difference between the temperature recorded at the start and end of melting. We compared our determined melting temperature with previous results for Cu ([11,28,29,30,31] and Au [13,29,31,32] (Figure 8) and found slightly higher values for Au and a trend in agreement with previous studies for Cu. The estimated error in the pressure scale was about ± 1 GPa.

### 3.3. Temperature-Dependent Electronic Thermal Conductivity of Cu and Au at a Fixed Pressure

**Figure 9:**
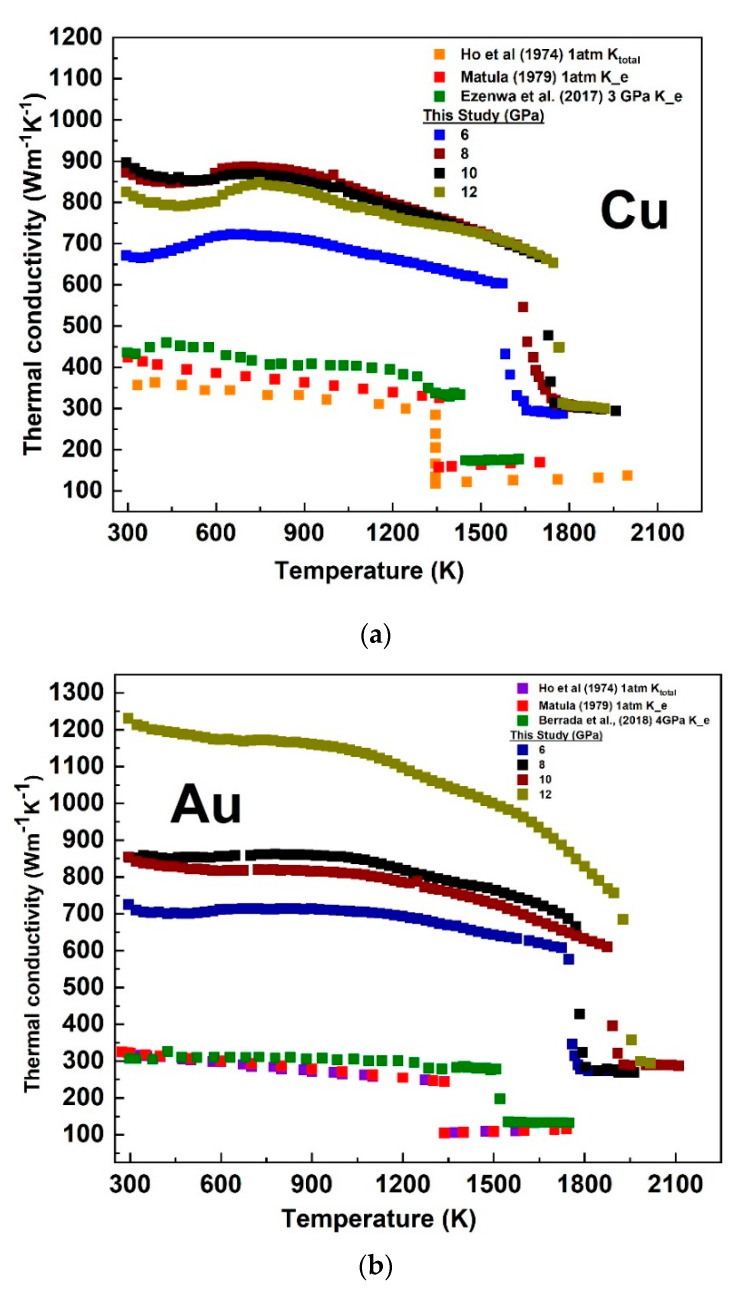
The temperature−dependent electronic component of the thermal conductivity of Cu (**a**) and Au (**b**) at pressures ranging from 6 to 5 GPa calculated from the electrical resistivity data, using the Wiedemann–Franz law with the Sommerfeld value of the Lorenz number. The data are compared to those calculated from the electrical resistivity reported at 1 atm and those measured at relatively lower pressure compared with the measured total thermal conductivity.

The difficulties in maintaining a well-controlled temperature gradient at high pressure and temperature conditions make direct measurements of thermal conductivities very challenging [33,34]. Hence, an indirect measurement is desirable. At 1 atm and above the Debye characteristic temperature for metals, the relaxation times associated with charge and thermal transport became comparable and tended to the Sommerfeld value (Lo = 2.445 × 10^–8^ WΩ/K2) of the Lorenz number [35,36]. To the best of our knowledge, the Lorenz number of Cu and Au at high pressures and high-temperature conditions has not been determined. The Wiedemann–Franz law (K_e_ = LT/ρ) allows for the electronic component of thermal conductivity, which is the dominant component in metals, to be calculated from the measured electrical resistivity (ρ) and temperature (T). This approach has been adopted by numerous studies (e.g., 18, [37,38,39,40,41,42]). Using the L_0_ value in the Wiedemann–Franz law, our calculated electronic thermal conductivity of Cu and Au at each fixed pressure was plotted as a function of temperature and compared with the calculated values from the 1 atm electrical resistivity data [21] and those measured at relatively lower pressure conditions for Cu [11] and Au [13]. We also compared our data with the experimental total thermal conductivity measured at 1 atm [43] (Figure 9). The general expected thermal conductivity decreasing with increasing temperature and increasing with increasing pressure in both the solid and liquid phases [35] agreed with our calculated values. There was no sign of pressure-induced structural transformation influence in the temperature-dependent thermal conductivity both in the solid and liquid state in agreement with the stability of the *fcc* structure at high pressure and temperature (see. [44]). The sudden decrease in the conductivity marked the solid–liquid transition in these metals.

## 4. Discussions on the Constant Resistivity along the Melting Curve of Cu and Au

The present study demonstrated that the electrical resistivity of Cu and Au along their respective melting curves was constant within an experimental uncertainty at higher pressure from 6 GPa up to our maximum investigated pressure of 12 GPa. The understanding of this phenomenon requires an understanding of how the antagonistic effect of pressure and temperature affects the Fermi surface of metals, in particular the simple metals. Compressing a metal increases the volume of the Brillouin zone in reciprocal space, which in turn increases the volume enclosed by the Fermi surface. For a cubic metal such as Cu, Ag, and Au, the Brillouin zone should ideally compress isotopically, and it is expected that a change in volume should cause no topological change in their Fermi surface [45]. On melting, the rigid-sphere theoretical model of the liquid metals demonstrated that the Fermi surface of many liquid metals with cubic or noncubic crystal structure was spherical and of a volume sufficient to accommodate all the valence electrons [46,47]. As highlighted by Ezenwa et al. [11], the spherical nature of the Fermi surface of the late transition liquid metals could generally suggest that thermal expansion could compensate for any possible distortion effect of pressure on the Fermi surface even for the nonisotropic compressible metals, at the onset of melting. If the antagonistic effect of temperature and pressure is limited to the above-highlighted effects, both could work in such a way to keep the resistivity constant along the melting curves. However, in simple metals, the increasing distance of separation between the *s* valence electrons and the filled *d*-band electrons with increasing pressure [48], may not be compensated with increasing temperature. The temperature-dependent investigation of the optical properties of liquid Cu indicates that the *d*-band is neither broadened nor shifted relative to the Fermi level by the melting processes [4]. The overall noncompensating antagonistic effects of pressure and temperature could lead to a decrease in the pressure-dependent resistivity along the melting curve due to an increase in nuclear screening [11]. While the above effect could be the reason for the decreasing resistivity of these metals along their pressure-dependent melting curves up to the investigated pressure of ~5 GPa [11,13], one could deduce that this effect could cease at higher pressure due to the establishment of equilibrium between the electron–electron repulsive and nucleus–electron attractive forces with increasing pressure. As such, increasing pressure may not have a significant effect in decreasing resistivity at much higher pressure. The constant electrical resistivity of Cu and Au along their respective pressure-dependent melting curve at a pressure range of 6–12 GPa (Figure 7) indicated that a state where the antagonistic effect of pressure and temperature could compensate each other could have been achieved. The decrease in pressure dependence at ~4 GPa at room temperature also pointed to the cessation of increasing nuclear screening with increasing pressure. At higher pressure above 5 GPa, the similarities in the observed constant resistivity along the melting curves of Pt [40,49,50] as well as Fe above ~5 GPa [18] suggest that partially unfilled late *d*-band metals could achieve Cu-like filled *d*-band state through the population of their *d*-band states by the promotion of *s* to *d* electrons through hybridization [14,15,16,17]. Thus, the constant electrical resistivity along the melting curve could be a universal theory that could be applicable to the transition metals, especially the late ones at higher pressure.

## 5. Conclusions

The temperature dependence of the electrical resistivity of high-purity Cu and Au have been experimentally measured at high pressures between 6 and 12 GPa and at temperatures of ∼150 K above melting temperature. Within error uncertainty, our results indicate that electrical resistivity of Cu and Au are constant along their respective pressure-dependent melting boundary from 6 GPa up to our maximum investigated pressure of 12 GPa, in agreement with the theoretical prediction. This was interpreted in terms of the saturation effect of the pressure-induced nuclear screening proposed to have caused the decreasing resistivity along the melting curve up to ~5 GPa by earlier studies. This also seems to correlate with a change in the resistivity slope at ~4 GPa at room temperature. Linear trends in resistivity as a function of temperature were observed at each fixed pressure in both the solid and liquid states and the solid-state dependence can be characterized by the Bloch–Grüneisen fit with variable “A” and “n” values. Within experimental uncertainty, the high-pressure melting temperatures of Cu and Au determined by the positive jump in resistivity fall within the range of melting temperatures reported by previous experimental and theoretical studies. Using the Wiedemann–Franz law alongside the Sommerfeld value of the Lorenz number, the electronic component of the thermal conductivity was calculated at each fixed pressure run. With increasing temperature, electronic thermal conductivity decreased in the solid state and tends to constant value in the liquid state.

## Figures and Tables

**Table 1:** Tabulated fitting parameters at various fixed pressures for Cu runs.

Cu		
Pressure (GPa)	“A” (µΩ·cm)	“n”
6	4.476	2.165
8	3.414	2.239
10	3.487	2.229
12	3.857	2.164

**Table 2:** Tabulated fitting parameters at various fixed pressures for Au runs.

Au		
Pressure (GPa)	“A” (µΩ·cm)	“n”
6	1.941	2.154
8	1.423	2.228
10	1.594	2.189
12	1.057	2.229

## Data Availability

The data is available within the article and Supplementary Materials and can be requested from the corresponding author.

## Supplementary Materials

The following figures are available online at https://www.mdpi.com/article/10.3390/ma14195476/s1, Figure S1. (A) Acquired real time graph of the temperature dependent electrical resistivity of Cu from room temperature up to about 150 K into melting at fixed pressure of 10 GPa. (B) Zoom−in of the of real time graph at high temperature and quench. (C) The plotted raw data of the measured voltage drop in both directions of the current Vs temperature, Figure S2. (A) Acquired real time graph of the temperature dependent electrical resistivity of Au from room temperature up to about 150 K into melting at fixed pressure of 6 GPa. (B) Zoom−in of the of real time graph at high temperature and quench. (C) The plotted raw data of the measured voltage drop in both directions of the current Vs temperature, Figure S3. The temperature-dependent electrical resistivity of solid and liquid of Cu measured at fixed pressure from 6 to 12 GPa, Figure S4. Graphs of temperature dependent electrical resistivity of solid Au at various fixed pressure, fitted with Bloch–Grüneisen formula, Figure S5. Graphs of temperature dependent electrical resistivity of solid Cu at various fixed pressure, fitted with Bloch–Grüneisen formula, Figure S6. Graphs of temperature dependent electrical resistivity of solid Au at various fixed pressure, fitted with Bloch–Grüneisen formula.
