# Supplementary material for: Electrical Resistivity of Cu and Au at High Pressure above 5 GPa: Implications for the Constant Electrical Resistivity Theory along the Melting Curve of the Simple Metals
Source: Materials (Basel). 2021 Sep 22;14(19):5476. doi: 10.3390/ma14195476 (PMC8509151; doi:10.3390/ma14195476)
Supplement: Supplementary file 1 [file materials-14-05476-s001.zip › materials-1355209-supplementary.pdf]

materials

Supplementary materials

Electrical Resistivity of Cu and Au at High Pressure above 5 GPa: Implications for the Constant Electrical Resistivity Theory along the Melting Curve of the Simple Metals

Innocent C. Ezenwa [1,2,*] and Takashi Yoshino [1]

[1] Institute for Planetary Materials, Okayama University, 827 Yamada Street, Misasa 682-0193, Tottori, Japan; tyoshino@misasa.okayama-u.ac.jp
[2] Now at the Earth and Planets Laboratory, Carnegie Institute for Science, Washington, DC 20015, USA
* Correspondence: iezenwa@okayama-u.ac.jp

See the Supplementary Material for the real time graphs and plots of acquired raw data. At fixed pressure of 10 GPa and 6 GPa for Cu and Au, respectively, we plotted the acquired sample voltage drop data versus temperature as shown in Figures S1 and S2. We show in Figures S3 and S4, the graphs of the temperature dependent resistivity of Cu and Au at each run fixed pressure. The solid-state resistivity dependence on temperature fitted with Bloch–Grüneisen formula are shown in Figures S5 and S6. The obtained fitting parameters were tabulated in Tables 1 and 2.

Citation: Ezenwa, I.C.; Yoshino, T. Electrical Resistivity of Cu and Au at High Pressure above 5 GPa: Implications for the Constant Electrical Resistivity Theory along the Melting Curve of the Simple Metals. *Materials* **2021**, *14*, 5476. https://doi.org/10.3390/ma14195476

Academic Editor: Jun Liu

Received: 9 August 2021
Accepted: 18 September 2021
Published: 22 September 2021

Publisher's Note: MDPI stays neutral with regard to jurisdictional claims in published maps and institutional affiliations.

Copyright: © 2021 by the authors. Licensee MDPI, Basel, Switzerland. This article is an open access article distributed under the terms and conditions of the Creative Commons Attribution (CC BY) license (http://creativecommons.org/licenses/by/4.0/).

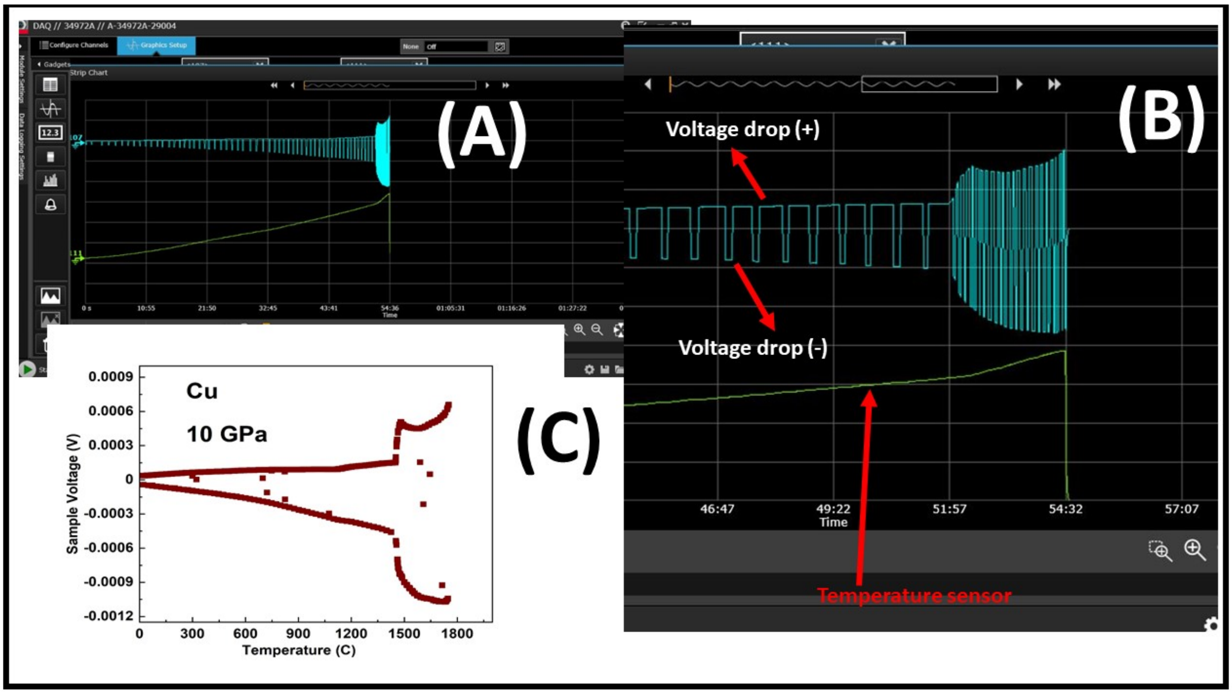

Figure S1. (**A**) Acquired real time graph of the temperature dependent electrical resistivity of Cu from room temperature up to about 150K into melting at fixed pressure of 10 GPa. (**B**) Zoom–in of the of real time graph at high temperature and quench. (**C**) The plotted raw data of the measured voltage drop in both directions of the current Vs temperature.

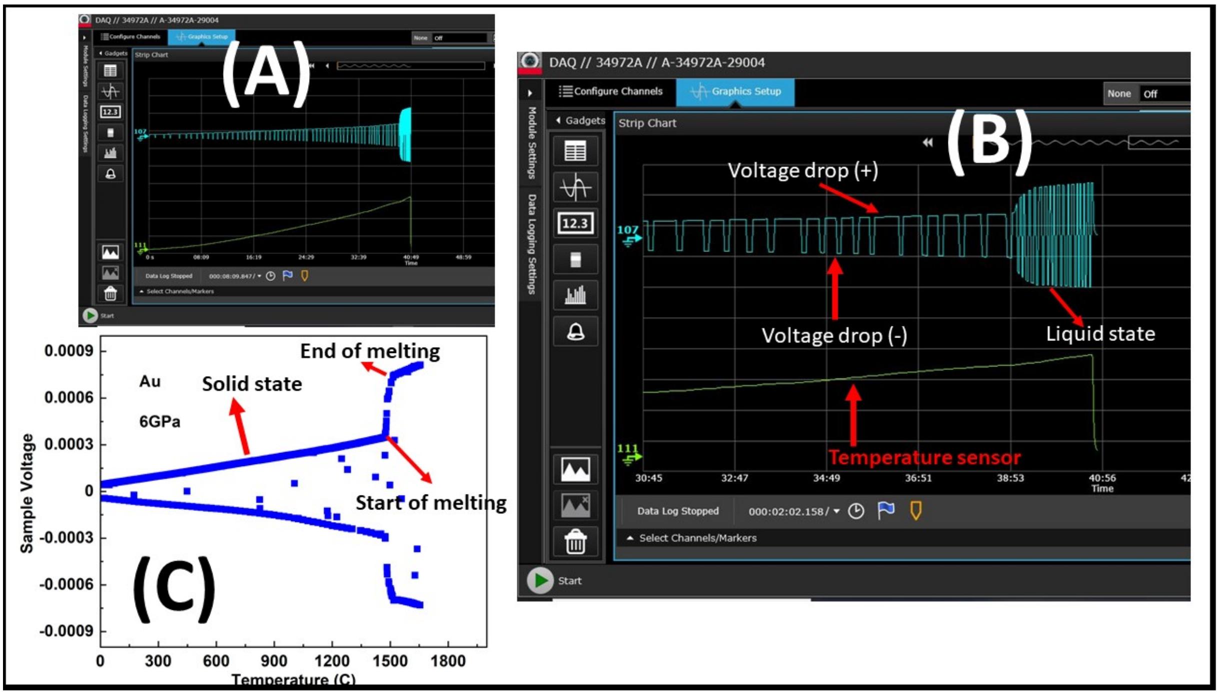

Figure S2. (**A**) Acquired real time graph of the temperature dependent electrical resistivity of Au from room temperature up to about 150K into melting at fixed pressure of 6 GPa. (**B**) Zoom–in of the of real time graph at high temperature and quench. (**C**) The plotted raw data of the measured voltage drop in both directions of the current Vs temperature.

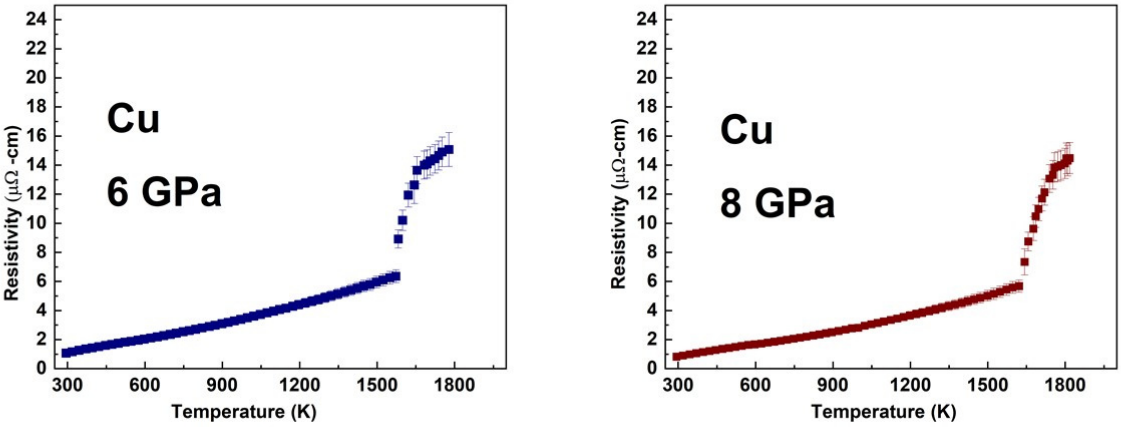

(**a**)

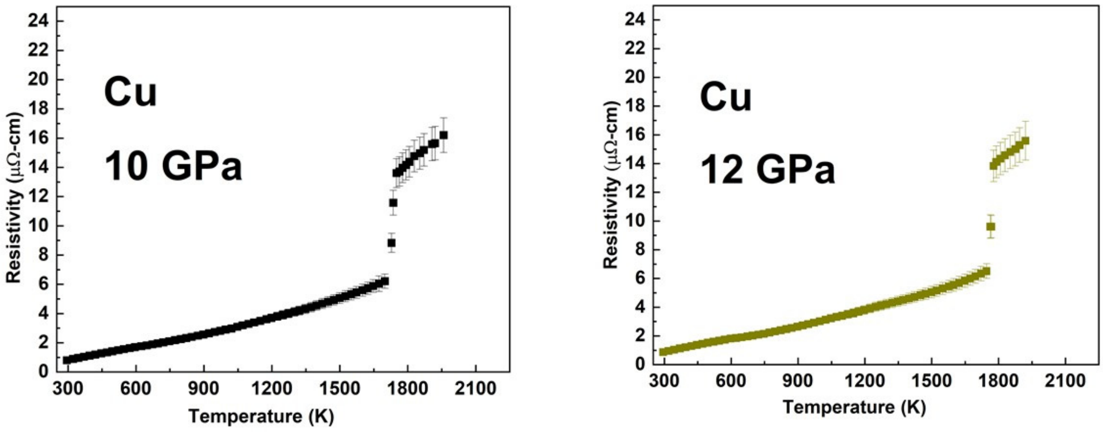

(**b**)

Figure S3. The temperature-dependent electrical resistivity of solid and liquid of Cu measured at fixed pressure from 6 to 12 GPa.

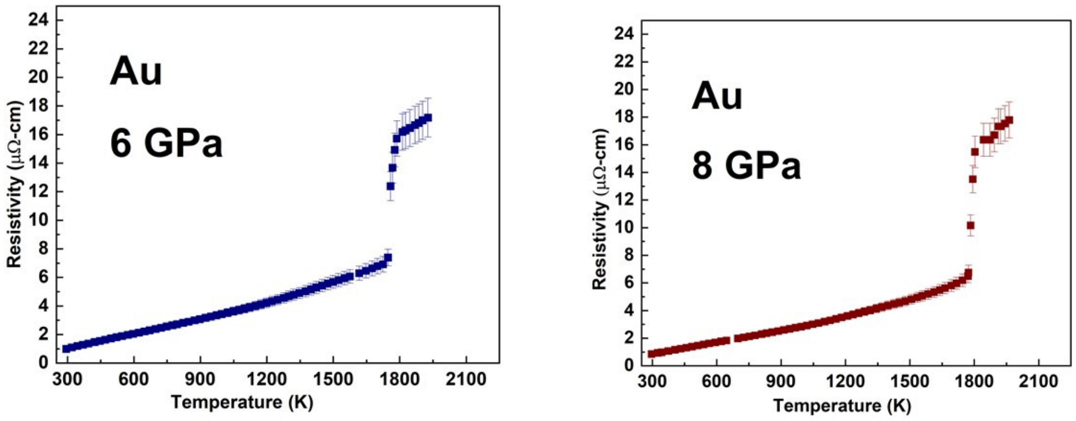

(**a**)

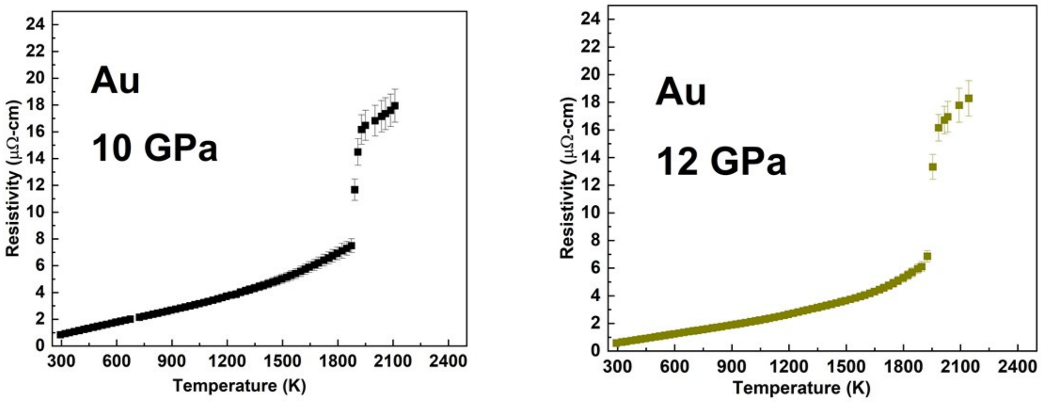

(**b**)

Figure S4. Graphs of temperature dependent electrical resistivity of solid Au at various fixed pressure, fitted with Bloch–Grüneisen formula.

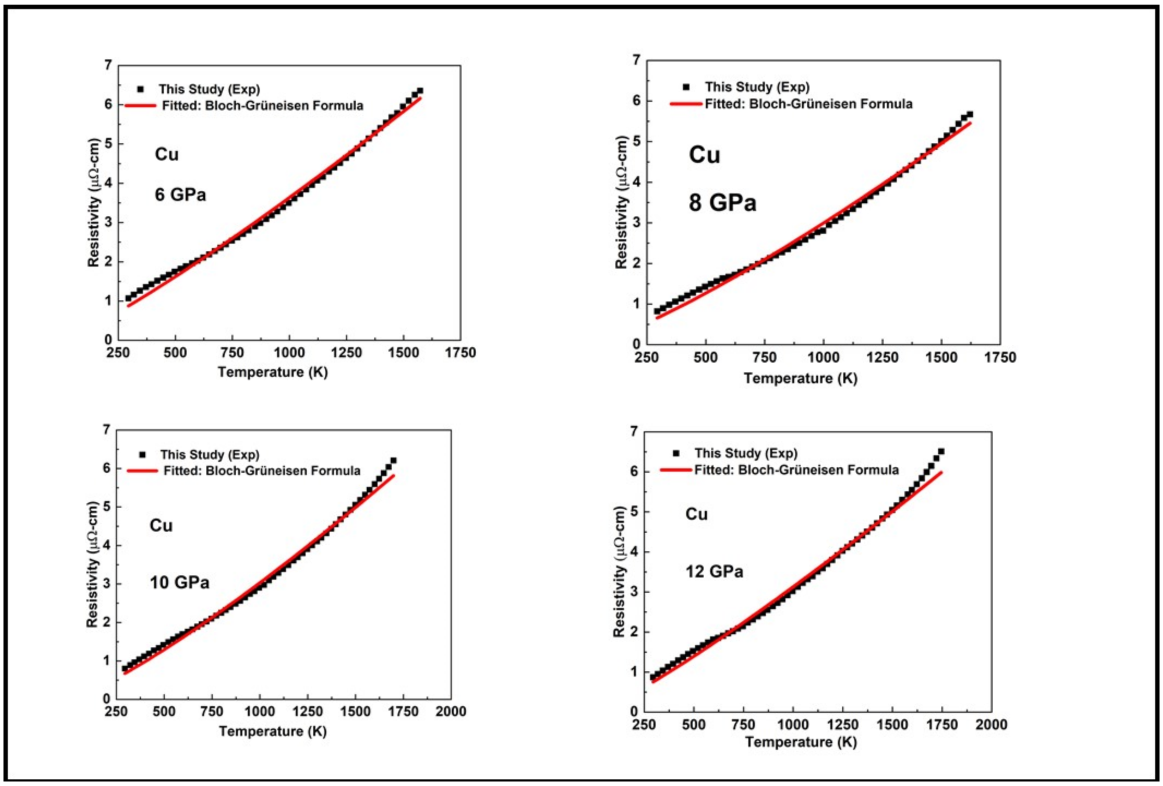

Figure S5. Graphs of temperature dependent electrical resistivity of solid Cu at various fixed pressure, fitted with Bloch–Grüneisen formula.

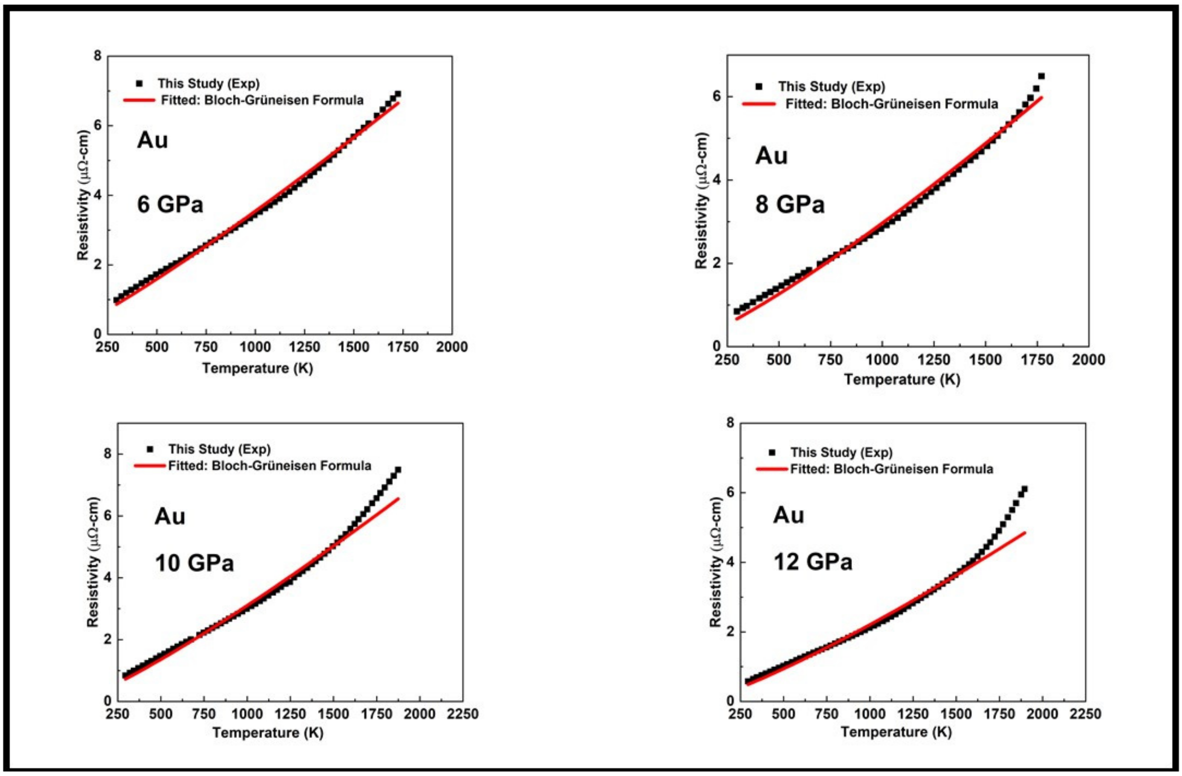

Figure S6. Graphs of temperature dependent electrical resistivity of solid Au at various fixed pressure, fitted with Bloch–Grüneisen formula.
